# Protocol of the HISTOTHERM study: assessing the response to hyperthermia and hypofractionated radiotherapy in recurrent breast cancer

**DOI:** 10.3389/fonc.2023.1275222

**Published:** 2023-12-19

**Authors:** Andreas R. Thomsen, Jörg Sahlmann, Peter Bronsert, Oliver Schilling, Felicia Poensgen, Annette M. May, Sylvia Timme-Bronsert, Anca-Ligia Grosu, Peter Vaupel, Jan-Olaf Gebbers, Gabriele Multhoff, Anne-Marie Lüchtenborg

**Affiliations:** ^1^ Department of Radiation Oncology, Medical Center - University of Freiburg, Faculty of Medicine, University of Freiburg, Freiburg, Germany; ^2^ German Cancer Consortium (DKTK), Partner Site DKTK-Freiburg, Freiburg, Germany; ^3^ Institute for Medical Biometry and Statistics, Medical Center - University of Freiburg, Faculty of Medicine, University of Freiburg, Freiburg, Germany; ^4^ Tumorbank Comprehensive Cancer Center Freiburg, Medical Center - University of Freiburg, Faculty of Medicine, University of Freiburg, Freiburg, Germany; ^5^ Institute for Surgical Pathology, Medical Center - University of Freiburg, Faculty of Medicine, University of Freiburg, Freiburg, Germany; ^6^ Pediatric Department, Black Forest Baar Clinic, Villingen-Schwenningen, Germany; ^7^ Medizinisches Versorgungszentrum Laaff, Freiburg, Germany; ^8^ Department of Pathology, Working Group Digital Pathology, University of Berne, Bern, Switzerland; ^9^ Center for Translational Cancer Research, Klinikum rechts der Isar, Department of Radiation Oncology, Technical University Munich (TUM), Munich, Germany

**Keywords:** breast cancer, hypofractionated radiotherapy, hyperthermia, predictive markers, longitudinal study, regression, recurrent breast cancer, re-irradiation

## Abstract

**Introduction:**

Breast cancer is globally the leading cancer in women, and despite the high 5-year survival rate the most frequent cause of cancer related deaths. Surgery, systemic therapy and radiotherapy are the three pillars of curative breast cancer treatment. However, locoregional recurrences frequently occur after initial treatment and are often challenging to treat, amongst others due to high doses of previous radiotherapy treatments. Radiotherapy can be combined with local hyperthermia to sensitize tumor cells to radiation and thereby significantly reduce the required radiation dose. Therefore, the combination treatment of mild local hyperthermia, i.e. locally heating of the tissue to 39-43°C, and re-irradiation with a reduced total dose is a relevant treatment option for previously irradiated patients. The mechanisms of this effect in the course of the therapy are to date not well understood and will be investigated in the HISTOTHERM study.

**Methods and analyses:**

Patients with local or (loco)regional recurrent breast cancer with macroscopic tumors are included in the study. Local tumor control is evaluated clinically and histologically during the course of a combination treatment of 60 minutes mild superficial hyperthermia (39 - 43°C) using water-filtered infrared A (wIRA) irradiation, immediately followed by hypofractionated re-irradiation with a total dose of 20-24 Gy, administered in weekly doses of 4 Gy. Tumor and tumor stroma biopsies as well as blood samples will be collected prior to treatment, during therapy (at a dose of 12 Gy) and in the follow-up to monitor therapy response. The treatment represents the standard operating procedure for hyperthermia plus re-irradiation. Various tissue and blood-based markers are analyzed. We aim at pinpointing key mechanisms and markers for therapy response which may help guiding treatment decisions in future. In addition, quality of life in the course of treatment will be assessed and survival data will be evaluated.

**Registration:**

The study is registered at the German Clinical Trials Register, Deutsches Register Klinischer Studien (DRKS00029221).

## 1 Introduction

Breast cancer is by far the most common cancer in women, accounting for 24.5% of all cancer cases in women. It is the most common type of cancer worldwide with a global incidence of 47.8/100000*year, and the leading cause of cancer-related deaths in women worldwide (1). Overall 5-year survival rates of breast cancer patients are high (above 80%) in many countries (e.g. 88% in Germany, 89% in the USA), albeit with pronounced geographical differences (2). Survival strongly depends on the tumor stage and subtype at diagnosis (3). Local recurrence occurs in <2-10% of all patients after breast-conserving surgery and radiotherapy, and in 4% of cases after mastectomy within five years (4–10). Patients who suffer from a loco(regional) recurrence have an increased risk of dying from breast cancer compared to those without recurrence (11, 12).

According to guidelines, radiotherapy is an important curative treatment option for breast cancer in addition to systemic treatment and surgery (13). The conventional fractionation scheme entails a daily dose of 1.8-2.0 Gy/5x per week, up to a total dose of 50 Gy, potentially augmented with a local boost of the tumor bed. Hypofractionation and ultrahypofractionation schemes, e.g. with a total dose of 40 Gy and single doses of 2.67 Gy/5x per week or 27 Gy in five fractions within one week, have also proven to be safe and effective. These alternative regimens offer the advantage of significantly reducing the burden of treatment for the patients due to shorter duration of the therapy (7–10). Approximately 60% of patients, regardless of tumor stages, receive radiotherapy (3). In addition, radiotherapy plays a critical role in palliative care, addressing the needs of patients with advanced local and metastatic disease. In instances of locoregional recurrent disease, radiotherapy is frequently combined with chemotherapy to enhance radiosensitivity and to reduce the risk of systemic progression (14).

Radiotherapy may be combined with mild locoregional hyperthermia (HT), i.e., the local heating of the tumor-bearing tissue to 39°C – 43°C (4, 15), administered before or after radiotherapy (16, 17). A non-invasive method for heating superficial malignant tissue is the application of the water-filtered infrared A (wIRA) irradiation to the treated tumor area (17). This method results in peak temperatures (~ 42°C) in the superficial layers of the skin. In 20 mm of tissue depth, wIRA can yield temperatures of approximately 40°C (17–19).

Mild hyperthermia causes radiation sensitization of tumor cells by (a) improving tissue oxygenation (20), (b) inhibiting DNA repair mechanisms and (c) stimulating the immune system without causing direct cytotoxic effects in normal tissue (15, 16). In addition, hyperthermia at temperatures >42°C for a longer exposure time leads to direct cytotoxicity of hypoxic and nutrient-deprived cells (21, 22). These effects on radiosensitivity have been demonstrated to be most prominent with a very short time interval between hyperthermia and irradiation (23). Therefore, we apply radiotherapy within <5 minutes after hyperthermia. This approach ensures the effective utilization of hyperthermia-associated tissue oxygenation. By employing this combination treatment strategy, local tumor control can be achieved with a significantly lower radiation dose than with radiotherapy alone, allowing effective and tolerable treatments even after multiple prior irradiations of the affected body region (16, 24–26). Hyperthermia combined with radiotherapy offers a treatment option for patients for whom neither surgery nor conventional fractionated radiotherapy is an appropriate or effective treatment option, and for patients with otherwise treatment-resistant tumors. The likelihood of achieving a complete remission with HT + re-irradiation is inversely correlated to tumor extent (16, 27). wIRA hyperthermia combined with a hypofractionated re-irradiation scheme of 5x 4 Gy/week, administered immediately after hyperthermia, has demonstrated very high success rates in breast cancer patients with recurrent disease (16, 19). At the same time, weekly treatment sessions may alleviate the treatment burden for patients (16, 19).

Predictive histologic and/or molecular markers, as used in systemic treatment (28), have not yet been identified for hyperthermia. The clinical potential of predictive markers for hyperthermia lies in patient-tailored therapy, which may include decision support for treatment escalation or de-escalation. Furthermore, the identification of predictive markers offers the opportunity to infer previously unknown mechanistic processes that are affected by the combination therapy. In particular, immunological processes are considered to be relevant (29).

In the HISTOTHERM study, clinical and histological tumor control rates in patients with locally advanced recurrent breast cancer will be analyzed over time, as well as the tolerability of the therapy and the quality of life of the patients during the treatment. In addition, our study intends to identify potential markers and processes which play a role in the combination treatment. These may help to define markers that could guide treatment decisions in the future. Finally, the study will allow the longitudinal observation of histological and cellular changes.

## 2 Methods and analysis

### 2.1 Study design

**Figure 1:**
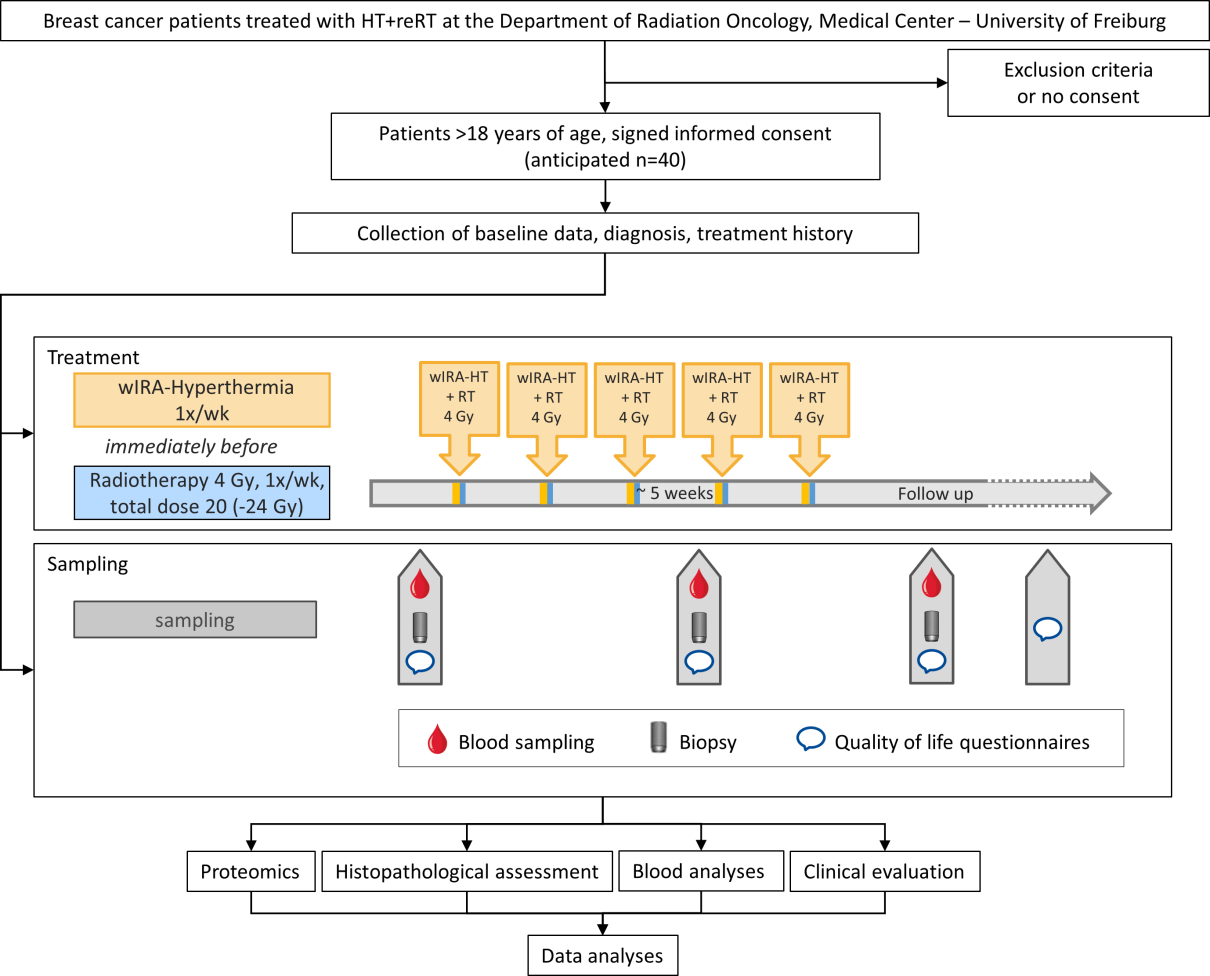
Schematic representation of the study design.

The HISTOTHERM study is a monocenter, prospective, non-interventional, longitudinal study taking place at the Department of Radiation Oncology, Medical Center – University of Freiburg. Trial sponsor is the Medical Center – University of Freiburg, Germany. Due to ethical reasons for non-existing further treatment options, patients are not randomized to treatment versus control conditions (30). The general scheme of the study is presented in **Figure 1**. Patient recruitment and treatment takes place at the Department of Radiation Oncology, Medical Center – University of Freiburg, Germany. Tissue samples are processed, and proteomic analysis is performed in the Department of Clinical Pathology, Medical Center – University of Freiburg. The Departments of Radiation Oncology and Pathology are part of the Comprehensive Cancer Center Freiburg (CCCF) and the diagnostics laboratory of the pathology is accredited according to DIN EN ISO/IEC 17020:2012. Blood is analyzed at the Klinikum rechts der Isar of the Technical University Munich (TUM), Germany. Patient recruitment is ongoing since 11/2019 and end of recruitment is expected by 2025. We wrote our report guided by the SPIRIT checklist (31).

### 2.2 Patients

Patients with local or (loco)regional recurrent (LRR) breast cancer after mastectomy, who present at the Department of Radiation Oncology at the Medical Center – University of Freiburg, Germany or in the interdisciplinary breast cancer tumor boards, will be screened for participation in the study. Patients with locoregional disease as classified according to the r-classes I-IV defined by Notter et al. (16) are eligible for the study. Central inclusion criteria is the indication for local mild superficial hyperthermia applied by wIRA combined with low-dose re-irradiation, i.e., patients with inoperable LRR that have a maximum depth extension of 20 mm. In addition, patients must (1) provide written, informed consent prior to enrollment and (2) be >18 years of age. Age <18 years, intolerance to local anesthetics, pregnancy, and lack of capacity to consent are exclusion criteria for this study. Distant metastases do not represent an exclusion criterion since they are common in patients with unresectable, locoregional recurrent breast cancer. Written informed consent was approved by the local ethics committee and will be obtained by the principal investigator from patients after sufficient time to read the patients’ information and discuss any questions. Informed consent to use biomaterial and associated data is collected using the informed consent form of the Center for Biobanking, Medical Center – University of Freiburg. Expected recruitment rates are high (>90%).

### 2.3 Interventions

Patients enrolled in the HISTOTHERM study are treated according to the standard operating procedure for the combination treatment of hyperthermia and radiotherapy at the study center. The area to be treated is irradiated with 20 Gy in single doses of 4 Gy every 7 days, immediately (i.e., within 2-5 min) after application of local hyperthermia, for 5 consecutive weeks in general. Radiation treatment planning is conducted based on computer tomography (CT) scans, with 8-10 mm expansion of clinical target volume (CTV) to obtain planning target volume (PTV). The choice between electron fields or volumetric modulated arc therapy (VMAT) plans is determined by the patients’ anatomy. To ensure precise positioning for VMAT plans, cone beam CT is used for correct patient alignment. Furthermore, surface optical scanning is employed to monitor and verify patient positioning accuracy. No RT-boost is applied. Re-planning is done if clinically indicated. Superficial hyperthermia is applied with thermography-controlled wIRA using the TWH 1500 wIRA-hyperthermia system (Hydrosun^®^, Müllheim, Germany) in order to locally heat the tissue volume to be irradiated to a temperature of 39°C-43°C for 60 minutes. Superficial tissue temperatures are thermography-monitored and automatically recorded as a treatment protocol. This documentation contains both temperature curves and thermographic images for each minute of treatment. Prior to the first treatment session, a punch biopsy is taken to confirm the initial diagnosis and to re-assess the histologic profile of the tumor. The biopsy is performed under local anesthesia using a 2.0 mm-3.0 mm disposable biopsy punch. The size of the punches is selected to obtain sufficient material without suturing the wound. Blood is collected from eligible patients using aseptic techniques in EDTA blood collection tubes (9 mL).

Treatment response is clinically evaluated and (photo-)documented weekly during the course of the treatment. At a dose of 12 Gy, a second punch biopsy is taken from the same tumor lesion for diagnosis of treatment response by evaluating tumor cell quantity and proliferation. Where applicable, another blood sample is taken. In case of a continuously high density of intact tumor cells and/or a high proliferation rate, a sixth treatment session may be considered to increase the total dose to 24 Gy. The success of the treatment is clinically evaluated at a follow-up appointment 6 weeks after completion of treatment. Another punch biopsy is taken to evaluate the histologic response and another blood sample will be drawn for further analysis. Clinical follow-up continues at 3-months intervals for one year, followed by 6 months intervals up to 5 years.

The total radiation dose administered may need to be adjusted on an individual basis. Patients who cannot continue treatment due to generalized progression or other reasons may receive a total dose of less than 20 Gy. When possible, these patients are monitored for treatment response and follow-up is performed as scheduled.

Furthermore, questionnaires are employed to assess patients’ individual quality of life before and during therapy and at the first follow-up appointment. This assessment is conducted using a standardized quality of life questionnaire sourced from the European Organization for Research and Treatment of Cancer (EORTC), EORTC QLQ-C30 (32), and an additional study-specific module on the tolerability of hyperthermia.

### 2.4 Data collection and analysis plan

#### 2.4.1 Baseline data

At enrolment, baseline demographic data and relevant clinical data about the course of the disease and treatment to date are retrieved from medical records and documented. These include date of diagnosis, localization and pathology of the primary tumor and of recurrences/distant metastases, previous and current therapies, and current disease stage and symptoms.

Study data are collected and managed using the Research Electronic Data Capture (REDCap) tool hosted at the Medical Center - University of Freiburg, Germany (33, 34). REDCap is a secure, web-based software platform designed to support data capture for research studies, providing (1) an intuitive interface for validated data capture; (2) audit trails for tracking data manipulation and export procedures; (3) automated export procedures for data downloads to statistical packages; and (4) procedures for data integration and interoperability with external sources (33, 34). Study records are pseudonymized and access to identifying information of the participants is strictly limited. Data will be retained for 10 years in accordance with policy.

#### 2.4.2 Local clinical and histological tumor response

The primary outcome of the HISTOTHERM study is the evaluation of local clinical and histological tumor response. Clinical tumor response is evaluated guided by the assessments defined by RECIST; notably complete response (disappearance of target lesion), partial response (>30% decrease), stable disease (< 30% decrease, <20% increase) and progressive disease (>20% increase) of the treated tumor (35). Since imaging diagnostics (CT/MRT) is not indicated as a standard therapy control during course of the treatment, treatment response is evaluated according to the investigator’s assessment. Clinical evaluation includes optical control, photographic documentation and palpation and is done during treatment and at follow-up visits six weeks after end of treatment followed by three-months intervals for the initial year and six-months intervals thereafter, for a cumulative duration of five years. Local clinical response is reported as discrete data (36).

Pathological regression is evaluated on Hematoxylin-Eosin (H&E) stained slides with Cytokeratin stained sections as reference according to the grading system of Miller-Payne in pre- and post-treatment samples (37). Frequency distribution and descriptive statistics are employed for the characterization of the treatment response. In order to compare molecular profiles and survival of different treatment responses, patients are grouped according to their clinical and histologic response. We will employ a generalized linear mixed model with Poisson distribution for evaluation.

#### 2.4.3 Identification of predictive markers and affected histological and cellular processes

Our overarching objective involves the discernment of tumor characteristics and the elucidation of a mechanistic basis underpinning responses to or resistance against the therapeutic intervention. Therefore, secondary outcomes of the HISTOTHERM study are the characterization of histologic and cellular differences within subgroups of patients to determine markers and key regulated processes involved in or activated by the combination therapy. To achieve these goals, comprehensive immunohistological and proteomic analyses on tissue and blood-based analyses will be undertaken.

Residual diagnostic tissue samples collected pre-treatment, during treatment (dose of 12 Gy) and at follow-up are used for analyses. Where available, tissue from the primary diagnosis is included in the analysis. Biopsies are routinely paraffin-fixed and embedded, followed by cutting of all required sections. After completion of routine diagnostics, study-specific immunohistochemical staining will be carried out on Formalin-fixed Paraffin Embedded (FFPE) tissue by trained staff of the CCCF biobank to obtain a comprehensive picture of the core processes involved.

Among the principal foci of investigation are the regulatory dynamics of the immune system, the mechanisms underpinning apoptosis and senescence. Approximately 20 different antibodies will be deployed for immunostaining of key proteins involved in those processes. Immunostaining will be performed in a standardized manner on multiple samples in parallel and will always include positive and negative controls to identify confounding staining effects. Subsequently, sections will be digitized and electronically archived on internal servers for further analysis.

Histologic sections are analyzed by trained staff who are blinded to clinical outcome. Depending on the marker protein under investigation, we will quantify and evaluate staining pattern, tissue distribution, intensity and number of positive and negative cells. Image analysis is supported by digital analysis tools, such as QuPath, which was developed specifically for pathological analysis (38). The tissue separation and cell detection tools are used for image classification and quantification of staining intensity, percentage of positive and negative cells and antibody distribution. Positively stained tissue structures are counted manually.

We will conduct a two-fold analysis of protein expression patterns, firstly focusing on alterations during the course of the treatment, and secondly comparing expression between patient groups displaying varying treatment responses. Histologic and clinical regression will be statistically correlated with the histopathologic findings. In pursuit of predictive histologic markers for therapy response, we will perform rate modeling using Poisson regression with offset and exploratory factor analysis. Cluster analysis will be employed to identify relevant biological processes in the tissue. The identified proteins and processes will lay the foundation for further investigations for therapy prediction.

Further, mass spectrometry-based proteomics is used to identify proteome alteration from the limited biopsy material. Proteome profiling by liquid chromatography – tandem mass spectrometry is performed essentially as described in our recent benchmarking study (39). From the biopsies before and if applicable during treatment and from the primary diagnosis, 1-2 sections of 10 μm section thickness will be prepared for proteomic analysis after micro- or macrodissection and measured on a Q-Exactive+ high performance mass spectrometer. Due to the scarcity of sample material, we focus on abundance proteomics. Data-independent acquisition with label-free quantification will be used.

We expect a proteome coverage in excess of 3000 proteins measured in our micro- or macrodissected tissue slides. For analyses, we will consider proteogenomic probing of common genomic alterations. Statistical evaluation will involve supervised and unsupervised approaches. Hierarchical clustering will be employed to identify proteomic subgroups, and algorithms that are tolerant for a low frequency of missing values will be considered. Protein expression will be associated with outcome data using multivariate Cox-type analyses, including consideration of boosting steps if required. We will also consider multivariate linear models, as highlighted by linear models of microarray analysis, including consideration of variable selection by tools such as elastic nets. Multiple testing correction will be considered. The proteomic profiles will be correlated with treatment stage and success to identify potential protein markers indicating a particularly good or poor response.

In order to identify systemic immunologic processes, EDTA blood (2 x 9 ml) will be collected from patients at the indicated time points for measurement of exosomal Hsp70 (40, 41) in the plasma, for enumeration of circulating tumor cells (40, 42, 43), and for a phenotypic characterization of lymphocyte subpopulations.

The major stress-inducible Hsp70 which is overexpressed in many highly aggressive tumor cells and metastases (44), promotes tumor growth due to its cytoprotective capacity (45). Tumor cells with a high Hsp70 expression frequently present Hsp70 on their plasma membrane (46, 47) and actively release Hsp70 in extracellular vesicles such as exosomes (48, 49). Therefore, Hsp70 will be measured in the blood of patients in the course of therapy by using the compHsp70 ELISA (40) which is able to quantify vesicular and free Hsp70. In contrast, normal cells contain Hsp70 in the cytosol but do not present it on the plasma membrane (50, 51). High levels of intra- and extracellular Hsp70 levels are associated with therapy resistance in different tumor types (52) and therefore, measurements of exosomal Hsp70 in the circulation during and after therapy might predict therapy response and can therefore be considered as a negative prognostic marker (44, 52).

The enumeration of circulating tumor cells (CTCs) which are considered as precursors of metastases are useful in predicting therapy resistance and overall survival in metastatic diseases (53) but a major limitation of this approach is the rarity of CTCs in the blood. Most antibody based selection approaches are based on antibodies directed against the epithelia cell adhesion molecule EpCAM (CD326) (54). However, it is known that following epithelial-to-mesenchymal transition (EMT) the expression of EpCAM is frequently downregulated on CTCs (55, 56), whereas the levels of membrane bound Hsp70 remains stably high (42, 57). Therefore, in this study we will comparatively examine the number of CTCs which are isolated on an EpCAM (epithelial CTCs) versus Hsp70 (epithelial and mesenchymal CTCs) antibody based bead approach in patients with advanced tumor disease in the course of therapy and in the follow-up period as a predictive marker for tumor aggressiveness and EMT.

Immunophenotyping of lymphocyte subpopulations in the peripheral blood will be performed to measure immunomodulatory activities of radiotherapy in combination with hyperthermia on the innate and adaptive immune system. It is well known that Hsp70 as one of the danger associated molecular patterns (DAMPs) can stimulate NK cell mediated immune responses (58). In a pilot study and in phase I and II clinical trials beneficial effects could be demonstrated by *ex vivo* Hsp70/IL-2 stimulated NK cells in patients with advanced colon and lung cancer (59–61). In this study we aim to measure the proportions of major lymphocyte subpopulations such as CD3+/CD4+ CD3+/CD8+ T helper and cytotoxic T lymphocytes, CD3+/CD94+ NK-like T cells, CD3+/CD4+/CD25+/FoxP3+ immunosuppressive T regulatory cells, CD19+ B cells, and CD3-/CD56+, CD94+, NKG2D+, NKp30+, NKp40+, CD69+ NK cell subpopulations in the course of therapy by multiparameter flow cytometry using fluorescence-labeled antibodies (59, 62). The aim is to identify an immune cell signature which might be able to predict clinical outcome.

#### 2.4.4 Quality of life

Enrolled participants are asked to complete paper questionnaires in person before the start of the treatment, before the 4^th^ treatment session at a dose of 12 Gy and at the first follow-up visit post-treatment. EORTC questionnaires have been designed, tested and validated to evaluate health-related quality of life in cancer patients and have been used widely used in clinical trials (32). To assess the individual quality of life during and after hyperthermia treatment, QoL questionnaires will be scored according to the guidelines of the quality of life group (36). The scores will be compared longitudinally to evaluate individual quality of life progression over time using a Mixed Model for Repeated Measures.

#### 2.4.5 Recurrence-free survival, distant-metastasis-free survival and overall survival

For the analysis of recurrence-free survival, distant metastasis-free survival and overall survival, the dates of loco-regional recurrences and distant metastasis and the time of death are recorded during follow-up. The date of loco-regional recurrence is defined as the date of detection of metastasis in the ipsilateral breast, nearby lymph nodes or chest wall. Recurrence-free survival is defined as the survival between start of treatment and the date of detection of locoregional recurrence. Distant metastasis-free survival is defined as the time between onset of treatment until the date of detection of distant metastasis. Overall survival is defined as the time between start of treatment and death. Correlation of clinical and pathologic outcome with recurrence-free survival, distant metastasis-free survival and overall survival are analyzed using Kaplan-Meier-plots and log-rank tests. Cox regression will be used to model the dependence on three additional variables.

### 2.5 Sample size calculation

Due to the exploratory nature of the study and for pragmatic reasons (63), 40 patients will be prospectively enrolled in the study. From each included patient, three biopsies will be taken and tissue from primary diagnosis or recurrence will be analyzed. Specimen staining is envisioned using approx. 20 antibodies. This results in 80 histological sections per patient to be processed and evaluated, and a total number of up to 3200 specimens to be examined. For proteome profiling our benchmarking study has highlighted that proteome alterations (e.g. between "responders" and "nonresponders”) are detectable with this sample size even in the presence of inter-individual proteome heterogeneity (39).

## Ethics statement

The study has been approved by the local ethics committee of the University of Freiburg, Germany (Ref 201/19).

## Author contributions

AT: Conceptualization, Funding acquisition, Methodology, Writing – original draft, Writing – review & editing, Supervision. JS: Conceptualization, Writing – review & editing, Methodology. PB: Conceptualization, Writing – review & editing. OS: Conceptualization, Writing – review & editing. FP: Conceptualization, Writing – review & editing. AM: Conceptualization, Writing – review & editing. ST-B: Conceptualization, Writing – review & editing. A-LG: Writing – review & editing, Conceptualization, Supervision. PV: Conceptualization, Writing – review & editing, Supervision. J-OG: Conceptualization, Writing – review & editing, Supervision. GM: Conceptualization, Writing – review & editing, Methodology. A-ML: Conceptualization, Writing – original draft, Writing – review & editing.
