# Erratum: Protocol of the HISTOTHERM study: assessing the response to hyperthermia and hypofractionated radiotherapy in recurrent breast cancer

**DOI:** 10.3389/fonc.2024.1414294

**Published:** 2024-04-19

**Authors:** Frontiers Production Office

**Affiliations:** Frontiers Media SA, Lausanne, Switzerland

**Keywords:** breast cancer, hypofractionated radiotherapy, hyperthermia, predictive markers, longitudinal study, regression, recurrent breast cancer, re-irradiation

Due to a production error, the incorrect affiliation was assigned to Andreas R. Thomsen, Oliver Schilling, Sylvia Timme-Bronsert, Anca-Ligia Grosu, Peter Vaupe, and Anne-Marie Lüchtenborg. The corrected author list and affiliations are listed below.

Andreas R. Thomsen1,2, Jörg Sahlmann3, Peter Bronsert4,5

Oliver Schilling2,5, Felicia Poensgen1,6, Annette M. May5,7

Sylvia Timme-Bronsert2,5, Anca-Ligia Grosu1,2, Peter Vaupel1,2,

Jan-Olaf Gebbers8, Gabriele Multhoff 9

and Anne-Marie Lüchtenborg1,2*

1Department of Radiation Oncology, Medical Center - University of Freiburg, Faculty of Medicine, University of Freiburg, Freiburg, Germany

2German Cancer Consortium (DKTK), Partner Site DKTK-Freiburg, Freiburg, Germany,

3 Institute for Medical Biometry and Statistics, Medical Center - University of Freiburg, Faculty of Medicine, University of Freiburg, Freiburg, Germany 

4Tumobank Comprehensive Cancer Center Freiburg, Medical Center - University of Freiburg, Faculty of Medicine, University of Freiburg, Freiburg, Germany

5Institute for Surgical Pathology, Medical Center - University of Freiburg, Faculty of Medicine, University of Freiburg, Freiburg, Germany

6Pediatric Department, Black Forest Baar Clinic, Villingen-Schwenningen, Germany

7Medizinisches Versorgungszentrum Laaff, Freiburg, Germany

8Department of Pathology, Working Group Digital Pathology, University of Berne, Bern, Switzerland

9Center for Translational Cancer Research, Klinikum rechts der Isar, Department of Radiation Oncology, Technical University Munich (TUM), Munich, Germany

The publisher apologizes for this mistake.

The original version of this article has been updated.

